# Effects of chest movements while sitting on Navon task performance and stress levels

**DOI:** 10.1186/s44247-023-00011-6

**Published:** 2023-04-13

**Authors:** Yoshiko Arima

**Affiliations:** Department of Psychology, Social and Psychological Research Center for Metaverse, Kyoto University of Advanced Science, 18 Gotanda-Cho, Yamanouchi Ukyo-Ku, Kyoto City, 615-8577 Japan

**Keywords:** Mobile sensing, Activity recognition, Machine learning, Remote work

## Abstract

**Background:**

This study explored physical activity during remote work, most of which takes place while sitting in front of a computer. The purpose of Experiment 1 was to develop a classification for body motion by creating a neural net that can distinguish among several kinds of chest movement. Experiment 2 examined the effects of chest movements on stress and performance on the Navon test to validate the model developed in Experiment 1.

**Method and results:**

The procedures for this study were as follows.

Experiment 1: Creation of the body movement classification model and preliminary experiment for Experiment 2.

Data from five participants were used to construct a machine-learning categorization model. The other three participants participated in a pilot study for Experiment 2.

Experiment 2: Model validation and confirmation of stress measurement validity.

We recruited 34 new participants to test the validity of the model developed in Experiment 1. We asked 10 of the 34 participants to retake the stress measurement since the results of the stress assessment were unreliable.

Using LSTM models, we classified six categories of chest movement in Experiment 1: walking, standing up and sitting down, sitting still, rotating, swinging, and rocking. The LSTM models yielded an accuracy rate of 83.8%. Experiment 2 tested the LSTM model and found that Navon task performance correlated with swinging chest movement. Due to the limited reliability of the stress measurement results, we were unable to draw a conclusion regarding the effects of body movements on stress. In terms of cognitive performance, swinging of the chest reduced RT and increased accuracy on the Navon task (β = .015 [-.003,.054], R^2^ = .31).

**Conclusions:**

LSTM classification successfully distinguished subtle movements of the chest; however, only swinging was related to cognitive performance. Chest movements reduced the reaction time, improving cognitive performance. However, the stress measurements were not stable; thus, we were unable to draw a clear conclusion about the relationship between body movement and stress. The results indicated that swinging of the chest improved reaction times in the Navon task, while sitting still was not related to cognitive performance or stress. The present article discusses how to collect sensor data and analyze it using machine-learning methods as well as the future applicability of measuring physical activity during remote work.

## Background

In the spring of 2020, when the COVID-19 pandemic began, Japanese universities shifted to online education according to governmental policies. However, adverse outcomes, such as an increased dropout rate, were found; thus, the Japanese government is currently instructing universities to reduce remote education. In the corporate sector, the adoption of remote work increased during the pandemic. However, some companies have begun scaling back from remote work and requiring employees to return to their offices. Thus, even though Japan ranked first in the world in the number of infected people under the seventh wave of the pandemic, in the winter of 2022, the rate of partially or fully remote work remained at 28.5% [1]. It is therefore important to determine healthy and efficient practice for remote work. For example, what activities promote or reduce performance in remote work? We aimed to create a self-monitoring tool instead of having management evaluate employees' performance. For this reason, we sought to categorize body movements with machine-learning methods that can be used with any smartphone. Such tools can be used by remote employees to obtain feedback regarding potential decreases in performance because these employees might not be aware of their stress levels.

### Impacts of sedentary behavior

The lockdown policies implemented to stop the spread of the COVID-19 pandemic caused health and psychological issues, including increased loneliness [2] [3] and increased anxiety and depression [4]. The lockdown-induced change in sedentary time (specifically, sitting) is the change that poses the most significant health risk.

The pandemic increased sedentary time (e.g., time lounging on the couch or bed) in daily life. According to [5] sedentary behavior increases all-cause mortality. Furthermore, there is a vicious cycle in which sedentary behavior induces a depressed mood, and the depressed mood further reduces physical activity[6]. According to a survey by KK Japan Innovation [7], 63.37% of university students showed symptoms of depression when Japanese universities conducted fully remote education. [8] found that greater physical activity and lower sedentary time were associated with reduced perceived stress after adjusting for sex, BMI, income, fruit and vegetable intake, alcohol consumption, and sleep quality.

To reduce sedentary behavior, various attempts have been made to prevent prolonged sitting. [9] compared groups of college students using sitting or standing desks and reported that the standing group had a more active lifestyle. One way to improve the mental health of individuals engaging in remote work and online learning is to use wearable sensors to measure physical activity. It is possible to identify signs of depression or activities that reduce depression, such as running, using data from wearable sensors. Data from such mobile sensors can be classified using deep learning for signal processing.

### Machine learning

In recent years, there have been large advances in the field of machine learning, and deep learning has become one of the most popular techniques for data analysis and prediction. Convolutional neural networks (CNNs; [10]) and long short-term memory (LSTM; [11]) models are representative deep neural networks. CNNs perform well for visual object classification, while recurrent neural networks (RNNs) are better suited for body motion recognition due to their ability to handle continuous data. LSTM was proposed as a method to solve the gradient loss problem of RNNs. LSTM models perform well with time-series data, such as text analysis. Deep learning algorithms are particularly useful for image classification and other computer vision tasks, but they can also be applied to signal data.

There are some differences between applying deep learning to image data and applying it to signal data that must be taken into account. When analyzing signal data with deep learning methods, preprocessing and feature extraction become more important than they would be with image data. Raw image data can often be fed directly into a deep learning network, but raw signal data are often noisy and variable, making it necessary to perform preprocessing before the data are ready for input into the network.

In this study, long short-term memory (LSTM) was selected for the classification of mobile sensor data. LSTM models are a type of recurrent neural network (RNN) that are particularly well suited for applications that require the analysis of sequences of data over time. This is because LSTM models are capable of learning long-term temporal dependencies in a sequence.

### Cognitive performance

While the negative effects of sitting on health are apparent, their effects on cognitive performance have been inconsistent [12]. examined the association between sedentary behavior and cognitive flexibility in adolescents and found that the association differs according to the type of sedentary behavior. Recreational screen-based sedentary behavior was found to have a negative association with cognitive flexibility, while educational (learning) sedentary behavior was found to have a positive association with executive control.

In the present study, we used the Navon (global–local) task to assess cognitive function [13]. [14] found that a positive mood improves (i.e., reduces) reaction times on global trials compared to local trials. The global–local processing mode is thought to be associated with meta-control functions that control processing tradeoffs, such as the one between speed and accuracy [15]. In the present study, we used shift trials, which involve switching between global and local trials, as an index of cognitive performance. Under more stressful conditions, the reaction time of shift trials in Navon tasks decreases because they require more attention than nonshift trials [16].

### Research question

This study aimed to identify indoor physical activities that promote or reduce cognitive function and stress. In particular, we compared the effects of sitting still with those of movements while seated during computer work. In particular, the study aimed to determine whether sitting still reduced cognitive performance compared to other sitting activities.

The purpose of Experiment 1 was to determine whether such subtle body movements could be classified. After creating a machine learning model to classify physical activity while seated, Experiment 2 examined which movements affected stress and cognitive performance during remote work. The goal of Experiment 2 was to use the model constructed in Experiment 1 to identify physical activities that negatively impact cognitive performance and increase stress levels while seated. Experiment 1 also included validation of the stress and cognitive performance assessments by conducting a preliminary experiment to gather a small amount of data before conducting Experiment 2.

## Methods

### Experiment 1: Constructing the model

#### Study setting

Most of the wearable sensors used in studies mentioned above were designed for specific purposes, such as card- or bangle-type sensors, which are connected via Wi-Fi. Considering technological trends and future applicability, we developed a machine learning model based on data from inertial sensors in smartphones. Smartphone sensors can collect a variety of data. We collected data from accelerometers and gyroscopes, which are the most commonly used sensors for analyzing body motion (e.g., [17, 18]).

The sensors were attached to the chest of participants to classify movements, assuming these movements were performed indoors during remote work or study. Chest movements are expected to be small compared to the arm and head movements involved in the operation of computers; thus, they reflect individual differences in movements while sitting.

For this purpose, the classification scheme of the Human Activity Recognition (HAR) study was modified to fit the remote-work situation; the HAR study equipped participants’ chests with wearable sensors to record physical activity during short events and basic activity architecture. Studies using support vector machines [19] have shown 96% recognition accuracy. Furthermore, [20] classified physical activities in daily life into six categories. (walking, climbing stairs, descending stairs, sitting, standing, and lying down). However, they reported significant misclassification of sitting and standing.

Reyes-Ortiz et al. [20] found that the time span of daily activities is approximately 2 to 5 s; machine learning should be able to distinguish movements within this time span. They also stated that sensors for obtaining 3-dimensional body movement data should have a sampling rate between 0 and 15 Hz. Because movements while sitting are typically subtle but continue over a lengthy period, a sampling rate that is too high will collect excessive data, reducing the feasibility of the study. Therefore, I used a technique that accurately captures minor motions while correctly differentiating among body movements at a sampling rate that collected a reasonable amount of data.

#### Participants

Eight participants (aged 19–62 years, six males and two females) were enrolled; five participated in the training session, and three participated in the preliminary experiment for Experiment 2. The participants signed an informed consent form. Before the experiments were conducted, the participants were informed about the APA's ethical standards for psychological research.

#### Procedure

##### Physical activity measurements

**Fig. 1:**
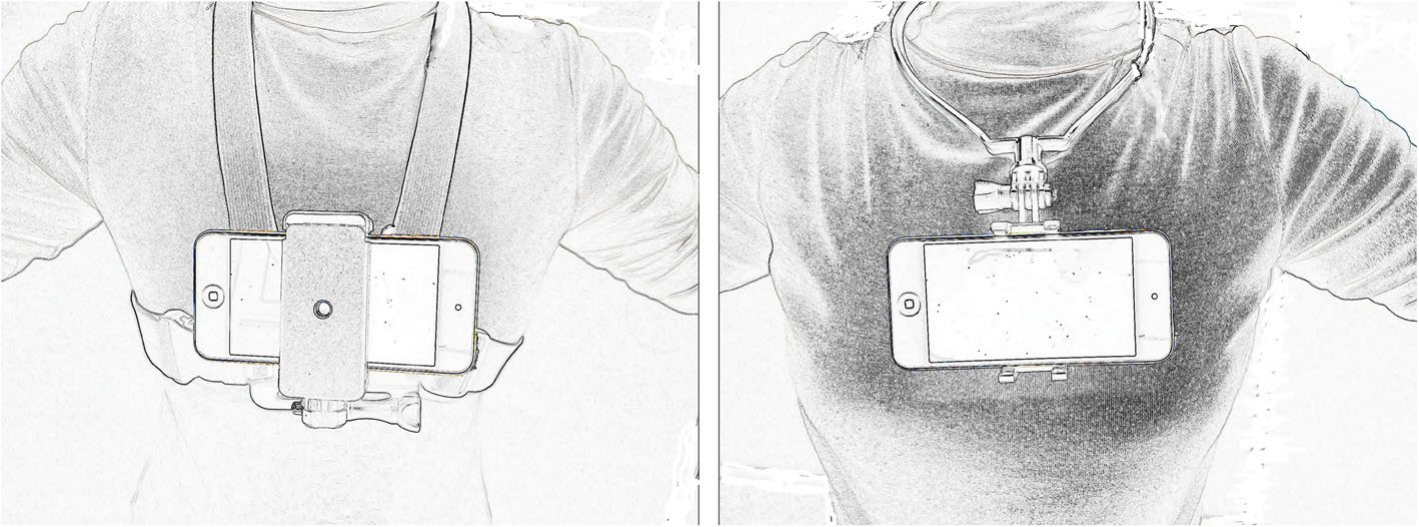
Attachments and the position of smartphones

The participants wore their smartphones horizontally at the center of the clavicle using a smartphone holder. Figure 1 (left) shows the smartphone holder used in the training session. For the preliminary experiment for Experiment 2, the participants used a different type of smartphone holder, as shown in Fig. 1 (right), for ease of use.

##### Training session: Supervised machine learning

Physical activity (10-Hz or 20-Hz data from the triaxial accelerometer and gyroscope) was recorded with the MATLAB mobile application. To collect labeled data for supervised machine learning, we recorded six movements by five participants (Participants A, B, C, D, and E). While the instructor counted 10 s for each activity, each participant was instructed to move as follows:

- (1) Walk
- (2) Stand up and sit down
- (3) Sit and rotate the upper body to the left and right
- (4) Sit and sway the upper body from side to side
- (5) Sit and rock the upper body back and forth
- (6) Stay still

We constructed LSTM models from the data obtained in the training session and cross-validated their accuracy rates.

The core components of an LSTM network are a sequence-input layer and an LSTM layer. The sequence-input layer is responsible for incorporating the time-series data into the network, while the LSTM layer is responsible for learning the long-term dependencies between the time steps in the sequence data over time. In this study, the author defined an LSTM network with 20 + 30 hidden units and a dropout layer with a dropout probability of 0.1. Dropout is a technique that helps prevent overfitting of models by randomly skipping some data during training. This ensures that the network does not simply memorize the training set and instead learns to recognize more general patterns in the data. The number of hidden units in the LSTM layer was relatively small; this number represents a trade-off between having enough memory to learn and avoiding overfitting. In this study, we aimed to develop a model yielding a sufficient accuracy rate while requiring less memory load.

##### Preliminary data for Experiment 2: Measuring body motion using the neural-net function in the training session

To validate the model developed in the training session, unlabeled sensor data from triaxial accelerometers were collected for 45 min from Participants F, G, and H, who did not participate in Experiment 1. Participant F stood and chatted with other students, Participant G sat and used a laptop computer at his desk, and Participant H sat for approximately half the time and stood for the other half of the time. After 45 min, the participants performed the Navon task and stress test as the preliminary experiment for Experiment 2. The procedure of the Navon task and stress test will be described in the sections on Experiment 2 below.

### Experiment 2: Testing the model

In Experiment 2, we used the model developed in Experiment 1 to measure subtle chest movements during seated desk work and examined their effects on cognitive performance and stress. As a measure of cognitive performance, we translated the Navon task for children [21–23], which is a script of the Inquisit test library made by Millisecond Software. This task involves a test of perceptual processing of global and local characteristics of stimuli using a circular or square graphic. As most global and local assessments employ English letters, we chose to use a test for children because our participants were Japanese.

Salivary amylase activity (SAA), which is employed as a measure of sympathetic activity to assess stress, has a well-established correlation with plasma norepinephrine levels. Thus, SAA can be used as an index of the norepinephrine concentration, which indicates sympathetic nervous system activation. In this study, we used the Nipro Cocoro METER, which measures SAA through a test chip inserted under the tongue of participants for 1 min [24]. Based on the results of Experiment 1, we measured SAA twice and used the larger value of two.

#### Participants

Thirty-four students from the psychology experiment class (21 males and 13 females, mean age: 20.46 years) were recruited from Kyoto, Japan. The participants were divided into two groups. One week before the experiment, the participants were familiarized with the procedure of measuring body movements with their smartphones and practiced the procedure.

On the day of the experiment, the participants were provided with a written explanation of informed consent, explaining that their participation was voluntary and that they could withdraw their consent at any time. Their rights to their data were explained as follows: "If you want to take this test but do not want your data to be used for analysis, please leave the parentheses in the ID number blank or use the number 99 when submitting your data. If you later decide that you do not want your data to be used for research, please contact the experimenter at that time."

After the informed consent information was distributed, the participants were directed to sign an informed consent form after the experiment was completed. The experimental participants were randomly given a card with an ID number, which they were asked to fill in when they submitted their data.

#### Procedure

##### Body movement measurements

The participants were seated approximately 1.5 m apart. The experimenter instructed the participants to download a file for their assignment to their laptops and prepare to start it. The assignment was about statistical analysis, but it was a relatively easy task for the participants to perform following the instructions. Smartphone holders were distributed, and each participant placed his or her smartphone in the holder as shown in Fig. 1 (right). After confirming that participants were ready, the experimenter instructed the participants to start the chest-movement measurements (on their smartphone) and to start the assignment (working on their laptop). After 30 min, the experimenter instructed the participants to stop the measurements and assignment. After stopping, the participants were asked to submit the triaxial acceleration data stored on their smartphones at their discretion.

##### Performance measurement

After 30 min of data collection with smartphone sensors, the experimenter asked the participants to complete the Navon task. The Navon task was administered with the Inquisit Web program launched on the internet browser of the participant PC. In the Navon task, participants observed the overall shape (circle versus square) and components composing the shape (small circles vs. small squares) of Navon figures. This task has three phases: (a) focus on the overall (global) shape; (b) focus on the detail (local) shape, and (c) mixed (randomized trials). The sequence of the trials is as follows: global (ten practice trials; 20 trials), local (10 practice trials; 20 trials), and mixed phases (12 practice trials of randomly selected global or local trials; 40 trials). In these tests, participants were instructed to use their mouse to click on one of the two options (circle or square) displayed beneath the target stimulus.

In this study, shift trials involved switching between global and local trials in the last 40 mixed trials (i.e., global trial → local trial). Nonshift trials were defined as trials in the same 40 trials that did not switch (i.e., global trial → global trial). The mixed test phases involved half shift trials and half nonshift trials, randomly distributed. Performance on the Navon task was assessed with two indices: the accuracy rate on the shift trials and the reaction time on the accurate shift trials.

##### Stress measurement

After the cognitive test was administered, SAA was determined with test kits. To prevent infection with SARS-CoV-2 (the virus that causes COVID-19), participants were instructed to perform the test themselves, fill in the two test results on the form, place the test paper in a plastic bag and return it to a designated place. The higher of the two test results was used as the stress index. The test results were submitted by participants on a voluntary basis.

At the end of the entire study, the experimenter explained the theoretical background of the experiment. Feedback was also provided to the participants by explaining the results of the experiment two weeks after it was conducted. Informed consent forms were submitted within these two weeks; 34 participants submitted consent documents. However, some of these participants did not submit smartphone data, collect sufficient data due to Wi-Fi malfunction, or had different sensor sampling rates. Data from these six participants were excluded as missing values, and data from 28 participants were included in the analysis. Two individuals did not submit SAA data because they failed to measure it, but their available data were included in the analysis.

Regarding additional data, we examined the validity of the SAA test for those willing to undergo a retest three weeks later. Ten participants performed SAA measurements and cognitive tests after climbing up and down stairs for 30 min.

## Results

### Experiment 1: Constructing the model

#### Training session

We applied CNN and LSTM networks to the data and used time-series partitions without shuffling the data. For cross-validation, data from four of the five participants (80%) were used as training data, and the remaining data were used as test data. Since all participants provided data for the test, network training was performed five times in the same manner, and the model was evaluated by averaging correct responses for the sets of data.

Only accelerometer data were used because the addition of gyroscope data reduced the accuracy of body movement identification in all datasets. The temporal signal was sampled from 10 to 128 Hz with a sliding window of 10 Hz (2 s) and a fixed width of 20 that allowed accurate identification of chest movements. Slice data with 50% overlap were used as training data, and each slice of data was subtracted from the mean for gravity correction and Fourier transformed. Data from each of the five participants was designated as the test data, and the remaining four datasets from participants were used as training data.

**Fig. 2:**
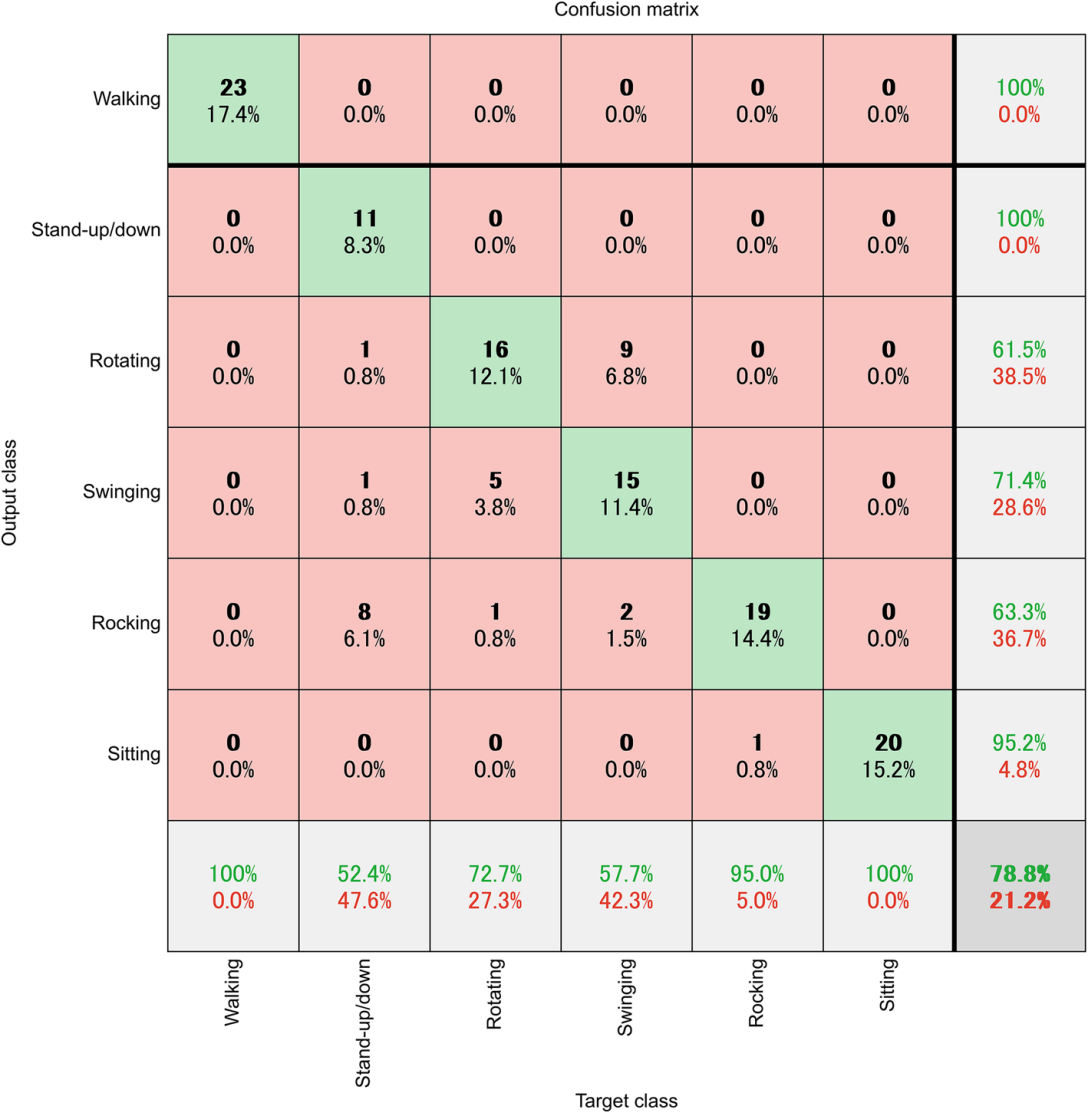
The confusion matrix of the lowest accuracy rate among the five participants is shown in Table 1. The test data were from Participant B, and the learning data were from Participants A, C, D, and E. The vertical categories are the classification results of the model, and the horizontal categories are the actual categories

The LSTM model had accuracy rates of 81.8% for Participant A, 78.8% for Participant B, 87.0% for Participant C, 83.3% for Participant D, and 88.1% for Participant E. The average accuracy rate was 83.8%. We further examined which categories had the largest errors. Figure 2 shows the confusion matrix of the lowest accuracy rate (78.8% for Participant B) among the five.

**Fig. 3:**
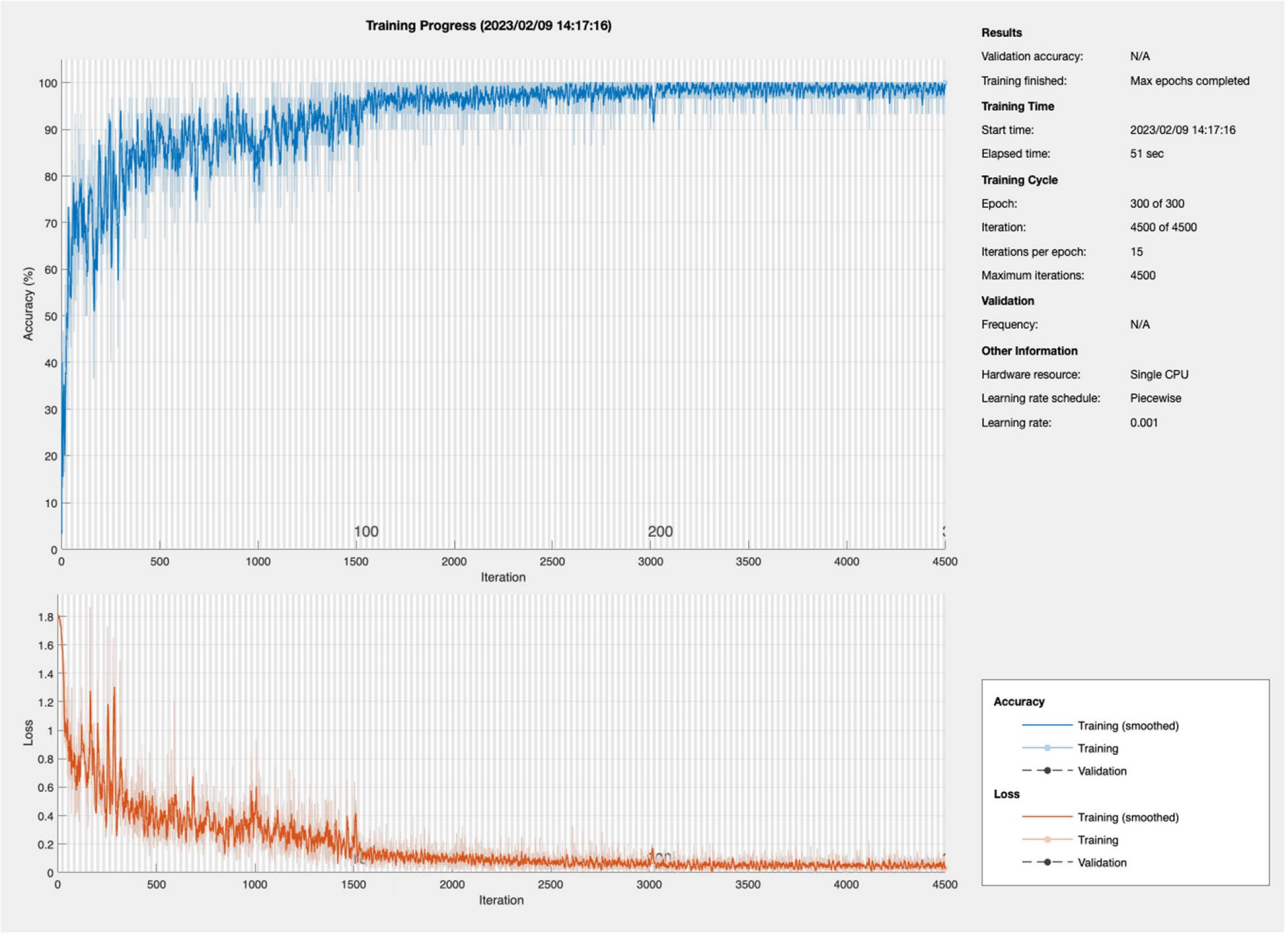
The learning process of LSTM using 5 participants data

The subsequent preliminary experiment for Experiment 2 used the LSTM model trained with data from all five participants. The parameters of this model were as follows: sequence-input layer = 3 (3-axis accelerometer), 1^st^ layer = 20, 3^rd^ layer = 30, output layer = 6 (6 categories), mini batch size = 30, and maximum epochs = 300. The solver was a stochastic gradient descent with momentum from the MATLAB program. The output metrics were as follows: iteration = 4500, last epoch precision = 100%, mini batch loss = 0.015, and learning rate = 0.001. Figure 3 shows the learning process of the LSTM model.

### Preliminary experiment for Experiment 2

**Fig. 4:**
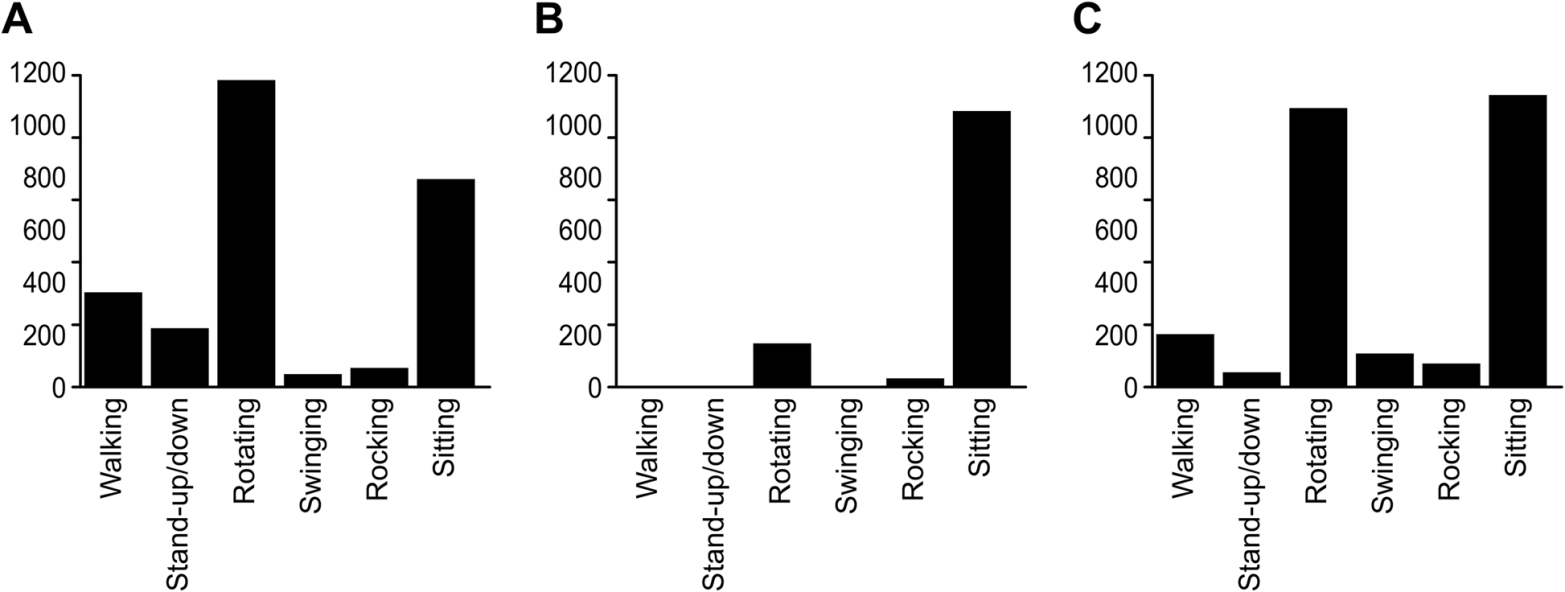
Identification counts of each body movement. Figure 3a (left) shows standing by Participant F, Fig. 3b (middle) shows sitting by Participant G, and Fig. 3c (right) shows standing and sitting by Participant H over 45 min

Figure 4 shows the counts of chest movements classified by the LSTM model created in Experiment 1 for Participants F (left figure; standing), G (center figure; sitting), and H (right figure; standing and sitting). These results reflected the subjects' behaviors, many of which were represented by only two categories, 3 (rotating) and 6 (stationary). The SAA data were 3.0 for Participant F, 2.0 for Participant G, and 2.0 for Participant H; these values are significantly lower than the values typically obtained (i.e., two-digit values) [25]. The reaction times on the Navon task were 1468.05 ms for Participant F, 1448.4 ms for Participant G, and 1157 ms for Participant H. Generally, these results suggest that our LSTM model, cognitive task, and SAA data were appropriate for our research purposes.

### Experiment 2: Testing the model

#### Preliminary analysis: Reaction time on cognitive tasks

**Fig. 5:**
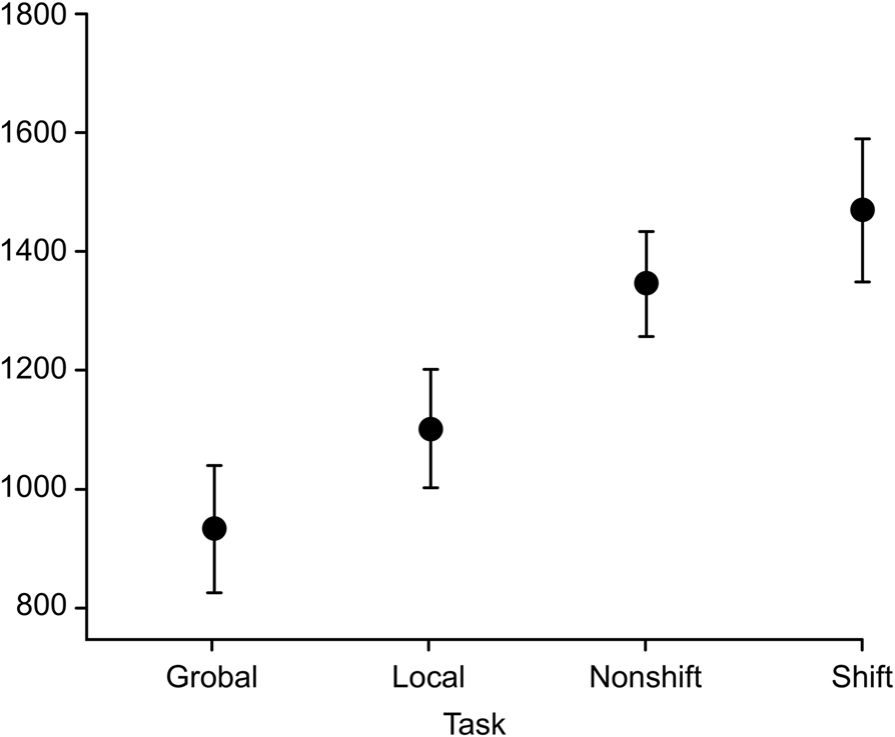
The vertical axis indicates the reaction time in milliseconds

Figure 5 shows the average reaction times for the four phases of the Navon task. A repeated-measures analysis of variance was used to examine whether there was a difference in these averages. Since the assumption of sphericity did not hold, Greenhouse‒Geisser correction was performed, and a significant effect was found (F(1.58, 42.72) = 22.63, *p* < 0.001, η^2^ = 0.46). The means (SDs) of the global, local, nonshift and shift trials were 932.83 (217.05), 1101.13 (302.79), 1345.168 (430.02) and 1468.83 (515.09), respectively.

Pairwise comparisons of the means revealed no difference between the nonshift and shift trials (Table 1). Although the Inquisit program by Sjöwall et al. [23] calculates the shit cost (shift cost = shift trial RT-nonshift trial RT), we used the mean of the reaction times on shift trials as a performance index because there were no significant differences between the shift trial RTs and the nonshift trial RTs.

**Table 1:** Comparison of shift and nonshift trials

Post Hoc Comparisons—Task						
		**Mean Difference**	**SE**	**t**	**p**_**bonf**_	**p**_**holm**_
Grobal	Local	-168.295	71.578	-2.351	0.127	0.042
	Nonshift	-412.336	71.578	-5.761	< .001	< .001
	Shift	-535.998	71.578	-7.488	< .001	< .001
Local	Nonshift	-244.042	71.578	-3.409	0.006	0.003
	Shift	-367.703	71.578	-5.137	< .001	< .001
Nonshift	Shift	-123.662	71.578	-1.728	0.527	0.088

*Note. P*-value adjusted for comparing a family of 6

#### Descriptive statistics

Table 2 shows the mean values for each measure obtained in Experiment 2. Sitting was the largest because the subjects were sitting in front of their laptops in the classroom during the 30-min measurement. Rotating was the next-largest category. SAA data for two participants was unavailable. The average accuracy rate for shift trials was high enough to obtain a reliable average reaction time.

**Table 2:** Descriptive statistics

	**Valid**	**Mean**	**Std. Deviation**	**Minimum**	**Maximum**
Walking	28	28.143	31.053	2	124
StandUpDown	28	38.214	24.745	7	79
Rotating	28	354.214	184.761	51	742
Swinging	28	44.536	21.384	9	88
Rocking	28	62.464	30.827	17	130
Sitting	28	1082.643	332.709	183	1489
Stress	26	25.462	15.103	3	68
Accuracy	28	0.968	0.08	0.6	1
ReactionTime	28	1468.83	515.088	711.417	3422.6

Regarding correlations among indices (Table 3), walking was strongly correlated with standing up and sitting down as well as rotating, rotating was correlated with rocking, and rotating appeared in sitting situations, as shown in Experiment 1. We discarded correlated indicators and therefore used rotating, rocking, and sitting as body movement indicators in the regression analysis. Concerning the mediator and outcome variables, a negative correlation was found between swinging and reaction time, and a positive correlation was found between the accuracy rate and SAA values. These variables were used in the mediation analysis.

**Table 3:** Correlation matrix among categories

**Variable**		**Walking**	**StandUpDown**	**Rotating**	**Swinging**	**Rocking**	**Sitting**	**Accuracy**	**ReactionTime**
1. Walking	Pearson's r	—							
	*p*-value	—							
2. StandUpDown	Pearson's r	0.598	—						
	*p*-value	< .001**	—						
3. Rotating	Pearson's r	0.467	0.647	—					
	*p*-value	0.012*	< .001**	—					
4. Swinging	Pearson's r	-0.105	0.166	0.341	—				
	*p*-value	0.596	0.398	0.076	—				
5. Rocking	Pearson's r	-0.188	0.166	0.377	0.352	—			
	*p*-value	0.338	0.398	0.048*	0.066	—			
6. Sitting	Pearson's r	-0.281	-0.303	-0.184	0.229	0.238	—		
	*p*-value	0.148	0.117	0.348	0.242	0.223	—		
7. Stress	Pearson's r	-0.062	0.178	0.047	0.182	-0.013	-0.036	—	
	*p*-value	0.77	0.395	0.825	0.384	0.949	0.865	—	
8. Accuracy	Pearson's r	0.012	-0.181	0.021	0.066	0.139	-0.158	-0.435	—
	*p*-value	0.954	0.365	0.918	0.742	0.49	0.43	0.03*	—
9. ReactionTime	Pearson's r	-0.011	-0.155	0.065	-0.39	-0.161	-0.061	0.206	0.245
	*p*-value	0.956	0.442	0.747	0.045*	0.424	0.761	0.324	0.209

Note: * *p* < .05, ** *p* < .01

### Mediation analysis

Due to the small sample size, the bootstrap method (2000 resamples) was applied to perform a mediation analysis. Physical movements during the 30 min (swinging, rotating, and sitting) were categorized as causes, while reaction time during shift trials was the mediating factor. The outcomes were stress and cognitive performance (accuracy on shift trials).

**Fig. 6:**
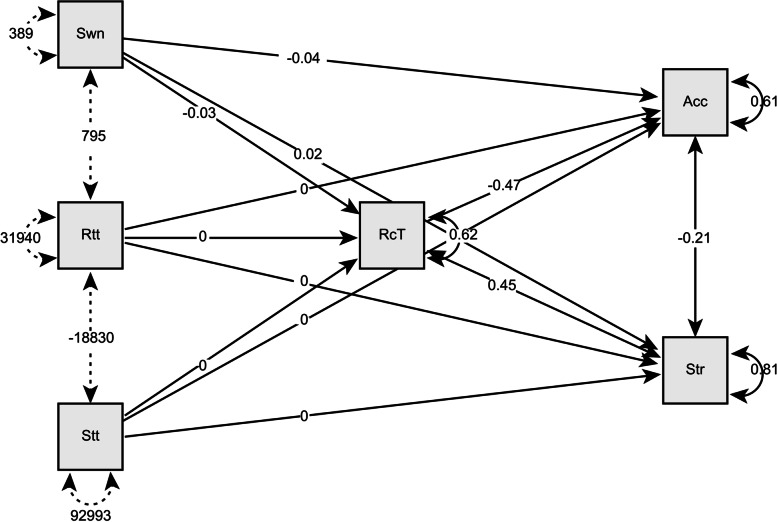
The numbers indicate the parameter’s standardized estimates

Table 4 shows the direct effects of the independent variables (chest movements) on the dependent variables (stress and cognitive performance). Table 5 shows the total effects. All paths in the mediation analysis, including indirect paths, are shown in Fig. 6.

**Table 4:** Direct effects of chest movement on cognitive performance and stress

Direct effects								
			**Estimate**	**Std. Error**	**z-value**	***p***	**Lower**	**Upper**
Swinging	→	Accuracy	-0.039	0.011	-3.628	< .001	-0.078	-0.012
Rotating	→	Accuracy	0.001	0.001	1.257	0.209	-0.002	0.004
Sitting	→	Accuracy	3.50E-04	5.98E-04	0.586	0.558	-6.89E-04	0.003
Swinging	→	Stress	0.022	0.012	1.752	0.08	-0.01	0.058
Rotating	→	Stress	-0.001	0.001	-0.83	0.407	-0.005	0.003
Sitting	→	Stress	-4.46E-04	6.93E-04	-0.644	0.519	-0.004	0.001

*Note.* Delta method standard errors, bias-corrected percentile bootstrap confidence intervals, ML estimator. NB: Not all bootstrap samples were successful: CI based on 1999 samples

**Table 5:** Total effects of the mediation analysis

Total effects								
							**95% Confidence Interval**	
		**Std. Error**	**Estimate**	**Std. Error**	**z-value**	**p**	**Lower**	**Upper**
Swinging	→	Accuracy	-0.024	0.01	-2.512	0.012	-0.042	0.008
Rotating	→	Accuracy	5.44E-04	0.001	0.491	0.623	-0.003	0.004
Sitting	→	Accuracy	1.16E-05	6.43E-04	0.018	0.986	-0.001	0.002
Swinging	→	Stress	0.008	0.011	0.717	0.474	-0.015	0.036
Rotating	→	Stress	-2.73E-04	0.001	-0.22	0.826	-0.005	0.003
Sitting	→	Stress	-1.26E-04	7.21E-04	-0.174	0.862	-0.003	0.002

*Note.* Delta method standard errors, bias-corrected percentile bootstrap confidence intervals, ML estimator. *NB * Not all bootstrap samples were successful: CI based on 1999 samples

The total R^2^ values for accuracy, stress and reaction time were 0.37, 0.15, and 0.36, respectively. Overall, swinging movements of the chest affected cognitive performance through RT. Swinging reduced the RT and increased accuracy; however, as shown in Fig. 5, the β value was small (standardized β = 0.015 [-0.003,0.054], R^2^ = 0.31).

### Additional analysis

To examine the validity of the SAA test used in this study, we retested the SAA and cognitive performance of ten participants after climbing up and down stairs for 30 min.

A comparison of the corresponding means revealed that the SAA level was 18.36 (SD = 11.55) after Experiment 2 (desk work), whereas the SAA level was 22.45 (SD = 16.95) after stair climbing. Although there was a trend toward higher SAA levels after stair climbing, the difference was not significant (t(10) = -0.95)). For the cognitive test, RT was lower after stair climbing, 1156.11 (SD = 248.51, t(9) = 2.66, *p* = 0.026), than during the main experiment (desk work), 1351.13 (SD = 243.7).

The SAA levels, which are predicted to be elevated by physical stress, were not significantly different after stair climbing than after desk work. Regarding the cognitive test, the effect of physical movement on reaction time was also confirmed in this additional analysis. From these results, the delay in the reaction time on shift trials in Experiment 2 was inferred to be due to cognitive fatigue rather than physical fatigue.

## General discussion

Experiment 1 showed that the 10-Hz accelerometer training data from four participants, measuring each movement for only 10 s, correctly classified 83.8% of the test data.

Seated status was not identified by lack of motion but by a combination of rotational chest motion and lack of other motion. Machine learning algorithms were able to identify movement categories from this study's small, short-sampled slice data. The LSTM model achieved good performance on accuracy and was able to handle sequential data. A sampling frequency of 10 Hz and a sampling slice of 1 s were sufficient to identify small body movements. Overall, Experiment 1 demonstrated that the participants' cell phones could be used as sensors during remote work.

Experiment 2 showed that swinging of the chest improved RTs in shift trials in the Navon task, while sitting still was not related to cognitive performance or stress. Since a correlation was found between RTs and an output category of the machine learning model, we believe that the machine learning model constructed and trained in this study successfully measured subtle chest movements. SAA values correlated with the accuracy rate, suggesting that stress decreased accuracy on shift trials, which require attention. However, we did not find a direct or indirect path between body movements and stress.

Additional analyses were performed to examine the validity of the SAA values. However, no significant differences were found even after stair climbing, which should have increased the physical load. Kreher et al. [26] found that SAA levels can prime the perception of unpleasant stimuli only when cortisol levels are high, suggesting that salivary amylase itself is an indicator related to cortisol levels rather than unpleasantness during task performance. To measure stress, a single indicator is not sufficient. A test battery is needed.

### Limitations of this study

Identification rates can be influenced by the environment, device used, and innate and acquired factors. In particular, the position of the wearable device may be important. In this study, the poor performance of the gyroscope could be attributed to the wearing position (on the chest). For example, the participant with the lowest identification rate, had standing up and sitting down events misidentified as rocking. It is natural for the upper body to move back and forth when standing or sitting.

It is possible that swinging of the chest was related to the participants’ arm movements. However, Mitra et al. [27] used VR and also found an association between lateral head sway and cognitive performance. For flanker tasks requiring similar attentional control, stress has also been reported to increase concentration in the short term [28]. For global–local tasks, [16] suggested that accessible information enhances the accessible reaction; thus, positive mood, not stress, may correlate with chest swinging.

Regarding the LSTM model, several limitations should be considered. For example, the performance of LSTM models can be sensitive to the choice of hyperparameters, such as the number of hidden units and the learning rate. If not properly supervised, LSTM models can overfit the training data and perform poorly on new, unseen data. Moreover, LSTM models can be difficult to understand and interpret, making it challenging to know how decisions are made and to diagnose problems. It is important to consider these limitations and carefully evaluate the performance of an LSTM model before deploying it in a real-world application.

## Conclusion

According to Experiment 2, sitting still did not impact stress levels or cognitive performance. On the other hand, subtle chest movements improved cognitive performance. Thus, sitting still does not negatively impact cognitive performance, but cognitive performance is compromised when small movements are decreased. However, since this study was conducted over a 30-min period, longer observation is necessary.

It was demonstrated that the model developed in Experiment 1 is applicable to such observations. In conclusion, LSTM models are a powerful tool for the classification of mobile sensor data. Using a Fourier transform to preprocess the data yielded highly accurate results.

This study imposed only a low burden of data processing from the user's cell phone since it did not require a high sampling rate to record subtle body movements. Additionally, the machine learning process required a low workload. These properties allow such models to subsequently adjust for individual differences to increase accuracy. In the future, we plan to apply the model from this study to virtual reality devices equipped with motion sensors to study the synchronization of remote worker movements.

## Data Availability

The deidentified dataset analyzed during Experiment 2 is available on OPENICPSR (openicpsr-178601). The data from Experiment 1 are available from the corresponding author upon reasonable request.

## Abbreviations

RT: Reaction time
Swn: Swinging
Rtt: Rotating
Stt: Sitting
RcT: Reaction time
Acc: Accuracy
Str: Stress
